# Advances and prospects of mucosal vaccination in the prevention and control of avian influenza

**DOI:** 10.3389/fimmu.2026.1766957

**Published:** 2026-02-05

**Authors:** Xianfeng Hui, Xiaowei Tian, Shihuan Ding, Ge Gao, Shuoxiang Gao, Aiping Sun, Tiesuo Zhao, Hui Wang

**Affiliations:** 1 Department of Immunology, School of Basic Medical Sciences, Henan Medical University, Xinxiang, China; 2 Henan Collaborative Innovation Center of Molecular Diagnosis and Laboratory Medicine, School of Medical Technology, Henan Medical University, Xinxiang, China; 3 Department of Pathogenic Biology, School of Basic Medical Sciences, Henan Medical University, Xinxiang, China; 4 Xinxiang Engineering Technology Research Center of Immune Checkpoint Drug for Liver-Intestinal Tumors, Henan Medical University, Xinxiang, China

**Keywords:** avian influenza virus, mucosal immunity, mucosal vaccines, respiratory tract immunology, viral shedding control

## Abstract

Avian influenza virus (AIV) poses a persistent threat to global poultry production and public health. Long-term immunization programs have established a foundational immune barrier, significantly mitigating the risk of highly pathogenic avian influenza outbreaks. However, the high mutability of AIV, complex biosafety requirements, and the accelerating scale of poultry production underscore the need for enhanced mucosal protection. Mucosal immunity represents a critical defense against respiratory virus invasion in poultry, rapidly mobilizing local antibodies, cellular immune responses, and innate defense mechanisms. Recent advances in mucosal vaccine platforms—including viral vectors, nucleic acid vaccines, nanoparticle-based delivery systems, and water- or spray-administered formulations—have demonstrated potential in poultry models to enhance local immune responses, reduce viral shedding, and improve herd-level immune uniformity. This review provides a systematic overview of the unique structural and immunological features of avian mucosal tissues, summarizes the latest developments in mucosal vaccine technologies, and discusses their potential applications and challenges within integrated avian influenza control strategies.

## 1 Introduction

Avian influenza virus (AIV), a highly mutable respiratory pathogen, exhibits a broad and complex global ecological distribution, particularly through a dynamic cross-host transmission cycle between domestic poultry and wild waterfowl (1, 2). Over the past decades, highly pathogenic H5 and H7 subtypes, as well as the low-pathogenic H9N2 subtype, have continuously driven viral evolution and dissemination, posing long-term threats to the poultry industry, biosafety systems, and public health (3, 4). Countries such as China have achieved substantial success in controlling certain highly pathogenic AIVs through sustained and systematic immunization programs, greatly reducing the likelihood of large-scale outbreaks and contributing to the stable development of the poultry sector (5–7). Nevertheless, from a global perspective, cross-species transmission, frequent gene reassortment, and ongoing antigenic drift remain major challenges (8, 9). The transmission chain between wild birds and domestic poultry is highly complex and dynamic, rendering AIV control efforts uncertain and demanding.

During AIV infection, the respiratory and intestinal mucosa serve as the primary portals of viral entry. Although avian and mammalian mucosal immune systems share fundamental structural principles, they differ markedly in anatomical organization, immune-associated tissues, and cellular composition (9, 10). In chickens, the nasal mucosa, tracheal-associated lymphoid tissue (TALT), and cecal tonsils together constitute an intricate mucosal immune network (11). AIV efficiently attaches to α2,3-linked sialic acid receptors on respiratory epithelial cells, enabling rapid replication and spread to deeper tissues; thus, effective immune recognition and containment at the mucosal level during the early stages of infection are essential to interrupt viral dissemination (12).

With advancing understanding of mucosal immunity, mucosal vaccination strategies targeting respiratory viruses have gained considerable attention (13). Recent progress in genetic engineering, nanodelivery platforms, biomaterials, and mucosal adjuvant development has enabled multiple mucosal routes—such as intranasal spraying, ocular administration, and drinking-water vaccination—to be applied in poultry (14, 15). A variety of innovative platforms, including viral-vector vaccines (e.g., NDV- or IBV-based vectors), nucleic acid vaccines, nanoparticle vaccines, conformationally stabilized HA (hemagglutinin) trimer antigens, and mucosal homing-enhancing adjuvant systems, have demonstrated promising results in avian models and, in some cases, industry-level application (16). These approaches not only induce systemic antibody responses but also establish durable mucosal immune memory in the upper respiratory tract, providing dual protection by limiting early viral replication and subsequent transmission (17).

It is noteworthy that avian mucosal immunity displays unique biological characteristics, such as developmental timing of immune organs, breed-specific differences in IgA secretion, and the crucial influence of microbial communities on mucosal immune maturation (18, 19). These features highlight the need to consider antigen delivery sites, adjuvant combinations, formulation stability, and immunization program optimization when designing mucosal vaccines (20). Moreover, accumulating evidence indicates that AIV can remodel the mucosal barrier by modulating interferon signaling, altering epithelial glycosylation patterns, and influencing mucin expression—further underscoring the importance of enhancing innate mucosal defenses (21).

Amid ongoing AIV evolution and increasing biosafety pressures in poultry production systems, developing next-generation mucosal vaccines to strengthen the “frontline immunity” of birds has become a key strategy for improving overall resilience in disease control (20). A systematic review of mucosal immune features, technological innovations in mucosal vaccine platforms, mechanisms of immune activation, and practical application prospects will contribute essential theoretical and practical insights for building more precise, efficient, and rapidly responsive immunization systems in poultry (22). Based on this rationale, this review will focus on avian mucosal immunobiology, novel mucosal delivery platforms, mucosal immune mechanisms, recent advances in vaccine technologies, and their implications for AIV prevention and control.

## 2 Mucosal pathogenesis of AIV infection

as a typical respiratory mucosal pathogen, the pathogenicity and transmissibility of AIV are tightly linked to the status of the host mucosal immune system (23). The upper respiratory and conjunctival mucosa constitute the initial interface where AIV encounters the host, and they represent the key anatomical sites for viral attachment, replication, and early dissemination (24). Understanding how AIV interacts with the mucosal environment is therefore essential for explaining the central role of mucosal immunity in limiting viral invasion, reducing viral shedding, and interrupting transmission within poultry populations. This section summarizes the mucosal pathogenic basis of AIV from the perspectives of tissue susceptibility, mucosal immune microenvironment, and viral immune-modulatory mechanisms, thereby providing a conceptual foundation for subsequent discussions on mucosal vaccine strategies.

### 2.1 Respiratory epithelium as the primary replication site of AIV

The avian respiratory epithelium is the critical biological site where AIV establishes initial infection, undergoes explosive replication, and initiates early spread. Its high susceptibility is mainly attributed to the abundant expression of α2,3-linked sialic acid receptors (α2,3-SA) on epithelial cells—the preferred binding moieties for avian-origin AIV (25). The nasal mucosa, tracheal epithelium, and primary bronchial mucosa exhibit both high receptor density and direct exposure to the external environment, making them the predominant anatomical regions where the viral replication cycle is first established under natural infection. Importantly, the conjunctival epithelium also expresses abundant α2,3-SA, rendering ocular exposure an underrecognized yet epidemiologically important portal of entry, particularly during outbreaks of highly pathogenic AIV (HPAIV) (26).

Upon binding to α2,3-SA, AIV rapidly enters epithelial cells via receptor-mediated endocytosis (27). Cleavage activation of HA by host proteases such as transmembrane protease, serine 2 (TMPRSS2) and human airway trypsin-like protease (HAT) is required for membrane fusion, while endosomal acidification facilitates the release of viral ribonucleoprotein complexes into the cytoplasm and subsequently into the nucleus, where transcription and replication occur (28). The high replication competence of AIV leads to the rapid accumulation of viral particles within infected regions, causing extensive epithelial cell death and detachment and resulting in the swift loss of ciliated structures. As a consequence, the mucociliary clearance system—a major physical barrier against inhaled pathogens—is severely compromised, permitting more efficient viral dissemination along the respiratory tract (29).

**Figure 1:**
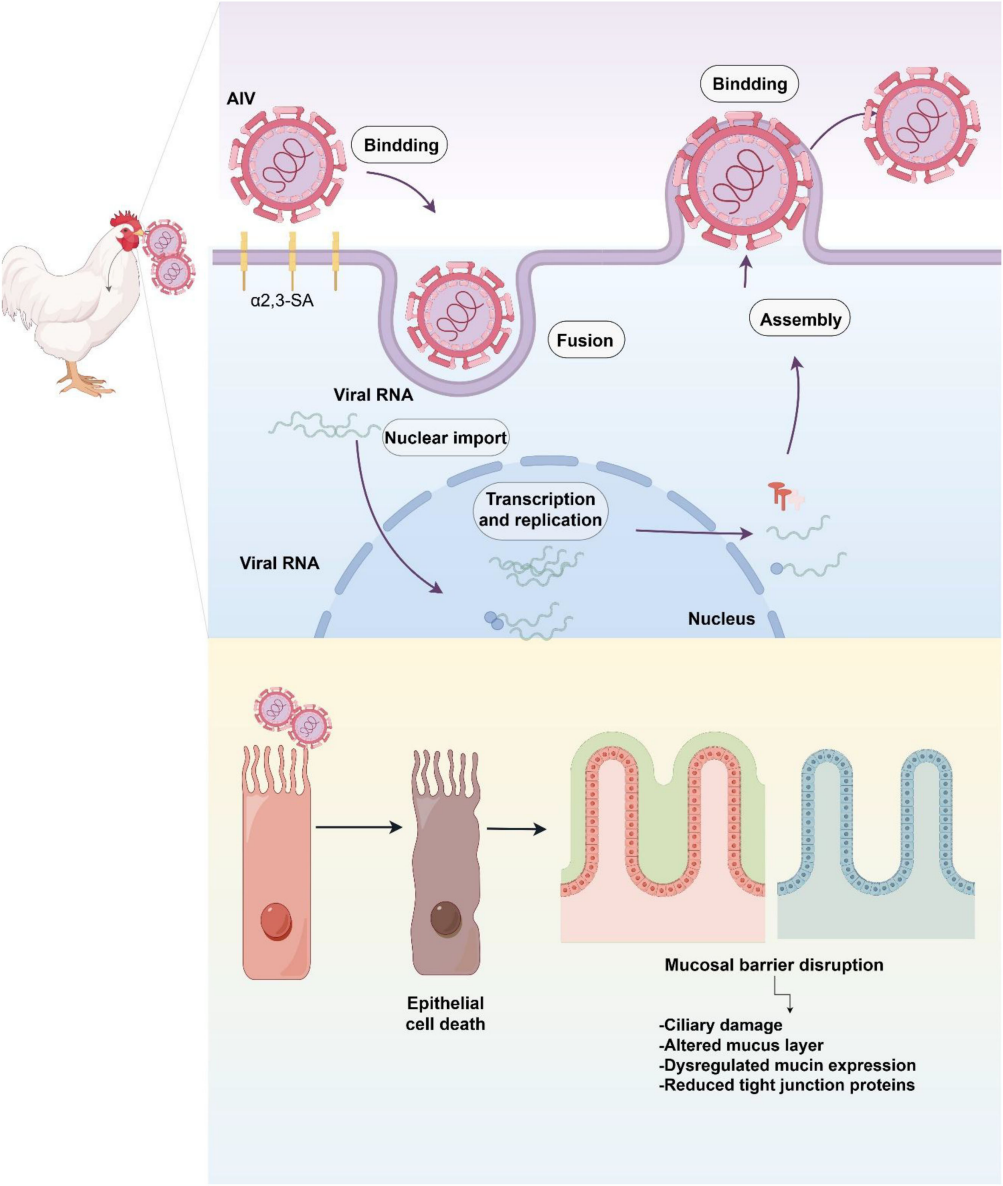
AIV-induced epithelial damage: from viral entry to impairment of mucociliary and tight junction integrity.

The disruption of the mucosal barrier extends beyond ciliary damage. AIV replication induces substantial alterations in the mucous layer, including disorganization of mucus architecture and dysregulated expression of mucins such as MUC5B (mucin 5B, oligomeric mucus/gel-forming) and MUC2 (mucin 2, oligomeric mucus/gel-forming) (30). Concurrently, tight junction proteins—including claudin-1, occludin, and ZO-1 (zonula occludens-1) —are markedly downregulated, leading to increased epithelial permeability (31). These combined effects weaken mucosal integrity and facilitate penetration of both viruses and secondary bacterial pathogens into deeper tissues, thereby escalating the risk of severe lower respiratory infection (**Figure 1**).

Furthermore, viral loads achieved within the respiratory epithelium are strongly associated with transmission efficiency among birds. Multiple animal studies have demonstrated that mucosal viral titers peak rapidly—within 24–48 hours post-infection—and correlate closely with subsequent levels of viral shedding (32). This early replication peak represents a major determinant of within-flock transmission velocity, transmission radius, and environmental viral contamination burden (33). Consequently, high-titer replication in the upper respiratory mucosa serves not only as a key node in infection dynamics but also as the starting point of large-scale transmission events.

Given these features, interrupting the first round of replication at the respiratory mucosal surface is considered one of the most effective strategies for breaking AIV transmission chains. Mucosal vaccines are specifically designed to exploit this principle by inducing robust local IgA responses, enhancing epithelial antiviral states, and promoting the formation of tissue-resident memory immune cells at the site of viral entry. By acting directly at the mucosal “portal of invasion,” such vaccines can block viral establishment before systemic replication ensues. Thus, the respiratory epithelium serves as both the biological foundation and the most critical target tissue for mucosal immunization strategies against AIV.

### 2.2 Role of (mucosa-associated lymphoid tissues) MALT in AIV infection

The avian mucosal immune system is highly developed and plays a central role in defending against early infection by AIV (34). The respiratory and ocular mucosa harbor a dense distribution of MALT, including the Harderian gland, conjunctiva-associated lymphoid tissue (CALT), bronchus-associated lymphoid tissue (BALT), and numerous scattered submucosal lymphoid follicles (35). Together, these structures constitute the first immunological surveillance network against invading pathogens and are particularly important along the mucosal routes through which AIV initiates infection (36).

A primary function of MALT is the rapid capture and presentation of viral antigens. The subepithelial compartments of the avian respiratory mucosa are enriched with dendritic cells (DCs) and macrophages capable of extending transepithelial dendrites to sample viral particles even before extensive replication occurs (37). Internalized antigens are processed and presented via MHC class II molecules to CD4^+^ T cells, thereby promoting the formation of local germinal centers and initiating antibody responses (38). Compared with mammals, birds possess more prominent BALT structures in the trachea and primary bronchi, making these tissues key sites for early T–B cell interactions.

Although γδ T cells are recognized in mammals as a crucial early immune barrier in epithelial mucosa—capable of rapidly sensing stress-induced signals following pathogen invasion and initiating localized antiviral responses—research on their counterparts in avian species remains relatively limited. At present, systematic empirical data are lacking regarding the distribution patterns, ligand-recognition mechanisms, and specific immunological functions of γδ T cells within the respiratory epithelium of poultry during early influenza virus infection (39, 40). Given the rapid-response properties and mucosal immunoregulatory roles demonstrated by γδ T cells in mammalian models, more comprehensive histological, immunophenotypic, and functional investigations are needed in avian species. Such studies are essential to determine whether γδ T cells serve a similar sentinel role during the initial stages of AIV entry and to assess their potential contribution to antiviral mucosal immunity in poultry.

**Figure 2:**
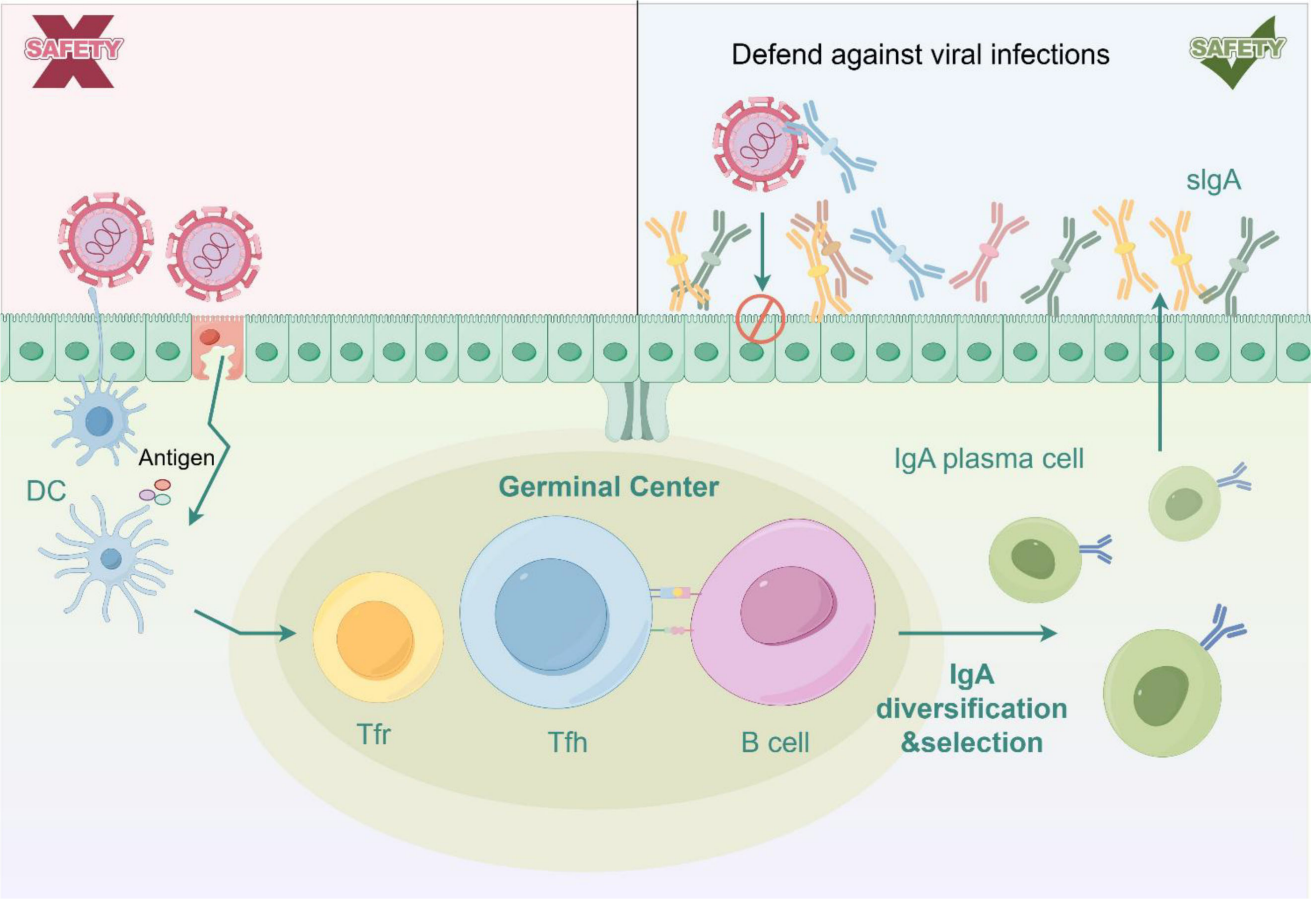
sIgA-mediated mucosal immunity and its blocking of viral transmission.

In humoral immunity, MALT serves as the major site for induction of secretory IgA (sIgA) in birds. Upon stimulation by viral antigens, local B cells undergo class-switch recombination within germinal centers, transitioning from IgM/IgD to IgA-producing phenotypes (41). With T cell help, these IgA^+^ B cells differentiate into plasma cells secreting large amounts of polymeric IgA, which is then transported through the epithelium via the polymeric immunoglobulin receptor (pIgR) (42, 43). At the mucosal surface, sIgA blocks the interaction between HA and sialic acid, prevents trans-epithelial viral spread, and neutralizes free viral particles. Notably, respiratory mucosal sIgA levels show a strong negative correlation with viral shedding, underscoring their importance in limiting transmission efficiency (**Figure 2**).

The Harderian gland holds a unique position in avian mucosal immunity. As the major immune organ draining the ocular-nasal region, it is particularly crucial for IgA induction following ocular or intranasal immunization (44). The gland not only produces high levels of sIgA but also supports long-term plasma cell residence, making it a principal anatomical basis for the protective efficacy of many mucosal vaccines delivered via eye-drop or nasal-drop routes.

Collectively, the MALT system—through antigen capture, coordinated T–B cell activation, sIgA production, and rapid participation of innate immune cells—effectively intercepts AIV at the mucosal surface before systemic infection is established. Its strategic location at the portals of viral entry makes MALT a critical anatomical and immunological target for the development of mucosal vaccination strategies against AIV.

### 2.3 Mucosal immune modulation by AIV

The infection and replication of AIV on mucosal surfaces depend not only on its receptor-binding properties but also on its ability to precisely modulate host mucosal innate immunity. Although the mucosa serves as the first line of defense against respiratory pathogens, AIV employs multilayered immune antagonism strategies to attenuate local antiviral defenses, thereby enabling rapid viral amplification in the upper respiratory tract and facilitating dissemination toward the lower airways.

A central determinant of viral immune evasion is the nonstructural protein NS1. This protein simultaneously disrupts multiple pattern-recognition receptor (PRR) pathways, including RIG-I/MDA5 and TLR3/7, thereby impairing epithelial sensing of viral RNA and markedly reducing the transcription of type I interferons (IFN-α/β) and interferon-stimulated genes (ISGs) (45, 46). NS1 can also bind the host E3 ubiquitin ligase TRIM25 to prevent ubiquitination of the RIG-I CARD domains, effectively blocking amplification of interferon signaling (47, 48). In addition, NS1 proteins from certain highly pathogenic H5N1 and H7N9 strains exhibit enhanced affinity for CPSF30, resulting in inhibition of host mRNA polyadenylation and processing, further suppressing the antiviral transcriptional response of mucosal epithelial cells (49). Together, these coordinated mechanisms allow AIV to efficiently dampen the antiviral state during the earliest stages of infection, laying the foundation for high-level replication.

Influenza virus HA and NA (neuraminidase) also contribute to the regulation of mucosal infection dynamics. The pH sensitivity of HA determines the efficiency of membrane fusion within endosomes; enhanced fusogenic activity under acidic conditions accelerates viral genome release and increases the rate of early replication (50, 51). NA cleaves sialylated glycoproteins abundant in the mucus layer and on epithelial surfaces, reducing mucus viscosity and limiting entrapment of virions, thereby allowing more efficient penetration of the mucus barrier (52). Some AIV strains possess NA with particularly strong enzymatic activity, significantly promoting lateral spread across the mucosal surface and increasing the number of initial infection foci.

The inflammatory response elicited by AIV plays a pivotal role in mucosal injury and disease progression. Following infection, epithelial cells, macrophages, and dendritic cells rapidly release proinflammatory cytokines such as IL-1β, IL-6, and TNF-α, which increase mucosal permeability and downregulate tight junction proteins, enabling deeper tissue invasion by viral particles and inflammatory mediators (53). Highly pathogenic H5 and H7 viruses strongly induce pyroptosis and ferroptosis, releasing large quantities of damage-associated molecular patterns (DAMPs) that amplify inflammatory cascades (54). This “inflammation–injury–replication” positive feedback loop constitutes a key pathological basis for acute mucosal destruction, vascular leakage, and systemic complications.

Humoral immunity, particularly mucosal IgA, is also critical for controlling AIV infection. Birds lacking functional sIgA are markedly more susceptible to infection, exhibiting uncontrolled early viral replication, significantly increased viral shedding, and prolonged viral clearance (55). Concurrently, mucosal barrier disruption and IgA deficiency contribute to respiratory microbiota dysbiosis, predisposing infected birds to secondary bacterial infections—most notably by *Escherichia coli* and *Staphylococcus* species—thereby exacerbating clinical severity and mortality (56). These observations underscore the indispensable role of mucosal barrier integrity and IgA responses in limiting initial viral invasion and breaking transmission chains.

Overall, AIV achieves efficient replication and transmission within the avian respiratory tract through a combination of innate immune antagonism, disruption of mucosal physical barriers, amplification of inflammatory injury, and evasion of humoral immunity.

## 3 Differences in immune protection between conventional vaccines and mucosal vaccines

Effective vaccination strategies against AIV must not only prevent clinical disease but also suppress viral replication and shedding, thereby reducing transmission within poultry populations. Conventional injectable inactivated vaccines and emerging mucosal vaccines differ fundamentally in their immunological induction sites, effector mechanisms, protective hierarchy, and flock-level epidemiological effects. These distinctions stem from the mucosal tropism of AIV and critically influence how each vaccination route controls initial infection and disrupts onward transmission.

### 3.1 Fundamental differences in immune induction sites and response characteristics

Conventional inactivated vaccines delivered intramuscularly direct antigen processing to systemic lymphoid organs such as the spleen and peripheral lymph nodes. This predominantly induces high serum IgY titers and circulating T-cell responses, which protect against systemic dissemination and severe pathology caused by virulent strains (e.g., H5) (57). However, because these vaccines do not deliver antigen to the upper respiratory mucosa—the primary portal of AIV entry—they elicit minimal local IgA responses, providing insufficient protection during the earliest stages of viral contact with epithelial surfaces.

**Figure 3:**
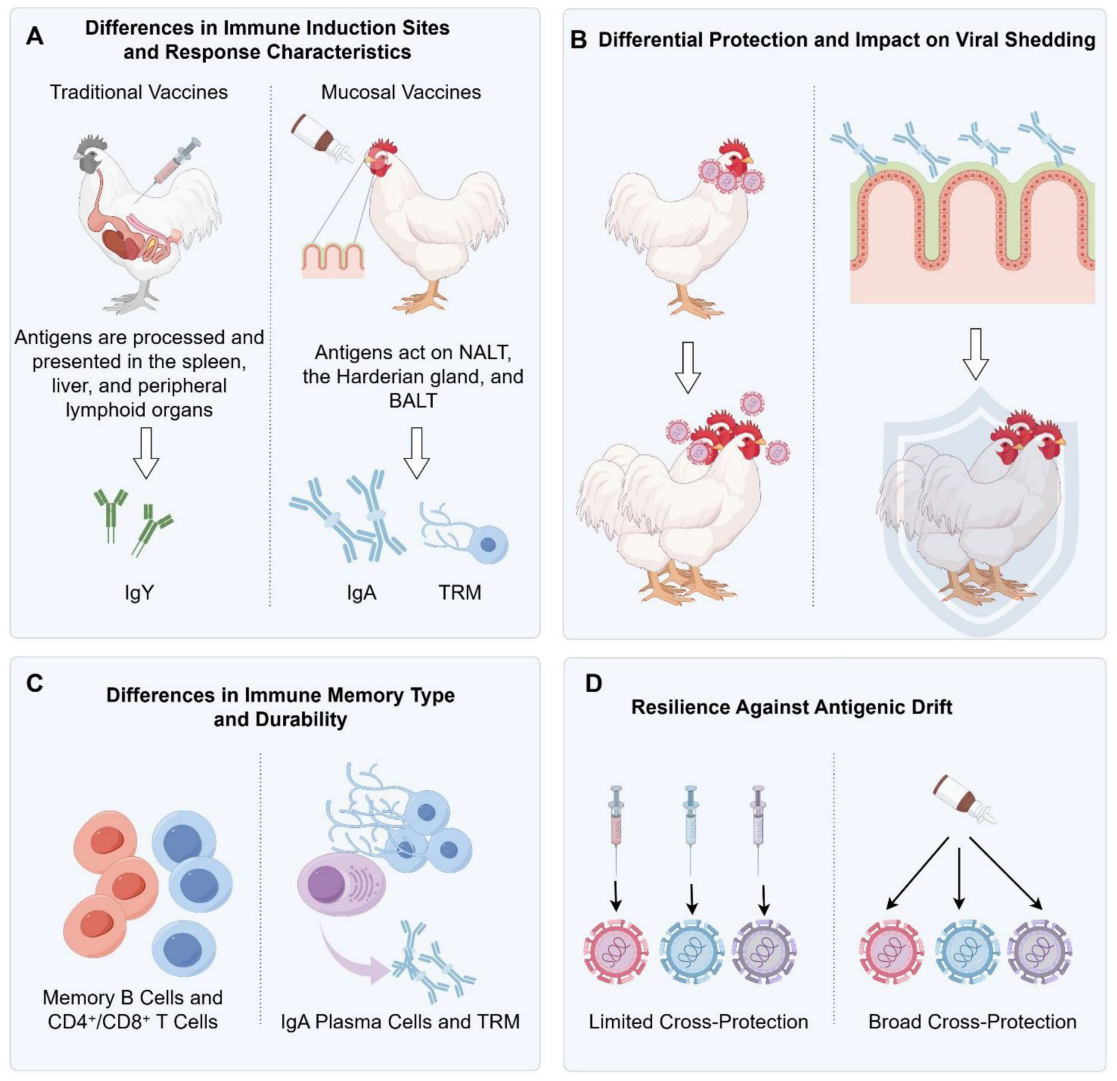
**(A)** Differences in immune induction sites and response characteristics. **(B)** Differential protection and impact on viral shedding. **(C)** Differences in immune memory type and durability. **(D)** Resilience against antigenic drift.

In contrast, mucosal vaccines administered via intranasal, ocular, spray, or drinking-water routes directly target mucosa-associated lymphoid tissues, including NALT, the Harderian gland, and BALT. Antigen is efficiently sampled by mucosal dendritic cells and resident phagocytes, driving the formation of IgA-secreting plasma cells, mucosal memory B cells, and tissue-resident memory T cells (TRM) (58) (**Figure 3A**). This localized immune architecture establishes a rapid-response barrier at the site of viral entry, conferring a substantial advantage for controlling AIV, which replicates predominantly in the respiratory tract.

### 3.2 Differential protection and impact on viral shedding

Traditional inactivated vaccines rely mainly on systemic neutralizing antibodies to limit viral dissemination and reduce lesions in major organs. However, they only weakly suppress early replication in the nasal epithelium, allowing birds—especially those infected with LPAIV such as H9N2—to continue shedding virus despite meeting serological protection thresholds (59). As a result, clinically protected birds can still contaminate the environment and sustain within-flock transmission.

Mucosal vaccines act earlier in infection by inducing strong local immunity. Secretory IgA blocks HA–sialic acid interactions, promotes viral aggregation, and prevents epithelial penetration, achieving rapid “immune exclusion” (60, 61). Concurrently, mucosal TRM cells eliminate infected epithelial cells within hours, preventing the establishment of substantial viral loads (62, 63). This confers a “pre-emptive blockade,” in contrast to the post-entry control typical of injectable vaccines.

Through enhanced sIgA responses, improved mucociliary clearance, and accelerated TRM-mediated cytotoxicity, mucosal vaccines sharply reduce both the magnitude and duration of viral shedding. Experimental and field studies show that shedding can decrease by several orders of magnitude, making onward transmission difficult in dense production settings (64, 65). Accordingly, mucosal vaccines more closely fulfill the role of true “transmission-blocking” interventions (**Figure 3B**).

Furthermore, effective control of viral shedding and transmission chains not only determines the success of disease control within poultry populations but also directly influences the risk of zoonotic spillover. Sustained viral shedding from the upper respiratory tract is considered a critical prerequisite for cross-species transmission of avian influenza viruses, particularly through airborne particles, contaminated dust, or close human exposure. By markedly reducing viral loads in the nasal cavity and trachea, mucosal vaccines can substantially decrease the amount of transmissible virus in the environment, thereby weakening the likelihood of viral spillover to humans at its source. Consequently, compared with conventional vaccination strategies that primarily aim to reduce clinical symptoms or mortality, mucosal immunization offers dual benefits within integrated avian influenza control programs: interrupting transmission within poultry flocks and lowering cross-species spillover risk. This dual functionality is of considerable importance for mitigating the potential emergence of future influenza pandemics.

### 3.3 Differences in immune memory type and durability

Immune memory induced by traditional vaccines is dominated by circulating memory B cells and CD4^+^/CD8^+^ T cells. The protective effect depends heavily on the persistence of serum antibody titers and antigenic match. When antibody levels wane or antigenic drift occurs, protection declines rapidly (66).

Mucosal vaccines, however, generate long-lived memory compartments directly within the nasal and tracheal lamina propria, including IgA plasma cells and TRM (**Figure 3C**). These cells operate independently of circulating immunity and provide immediate local protection upon re-exposure. Such “on-site immune memory” contributes substantially to durable inhibition of viral replication and shedding.

### 3.4 Resilience against antigenic drift

AIV—particularly H9N2 and certain H5 subtypes—undergoes rapid antigenic drift in poultry populations, necessitating frequent seed strain updates for traditional inactivated vaccines (67). Because these vaccines rely primarily on systemic neutralizing antibodies, cross-protection against drifted or heterologous strains is often limited, resulting in “protection lag” during active circulation.

Mucosal immunity exhibits greater resilience to antigenic variation. sIgA possesses broad binding activity and recognizes relatively conserved HA domains; even when epitopes mutate moderately, sIgA often retains capacity to block infection (68). Its multivalent polymeric structure also promotes viral aggregation and immune exclusion, limiting early viral expansion at the mucosal surface and thereby reducing shedding (**Figure 3D**).

Furthermore, mucosal vaccines effectively elicit TRM responses directed against conserved internal proteins such as NP and M1 (69–71). These TRM cells are strategically positioned at the infection portal and swiftly eliminate infected cells before extensive viral replication occurs, providing inherent cross-subtype protection. Concurrent upregulation of interferon-stimulated genes (ISGs) and antiviral effector proteins enhances innate resistance to drifted strains (72).

Collectively, through sIgA-mediated cross-protection, TRM recognition of conserved antigens, and heightened innate antiviral states, mucosal vaccines deliver broad, stable, and durable immunity—an advantage highly relevant for suppressing shedding and interrupting transmission in the context of rapid AIV evolution.

### 3.5 Divergent impacts on herd immunity

Under modern high-density poultry production systems, vaccine performance must be evaluated not only by individual-level protection but also by its capacity to interrupt transmission at the population level. Although traditional inactivated vaccines induce strong systemic antibody responses and greatly reduce disease severity and mortality, their limited ability to suppress respiratory and gastrointestinal shedding means that vaccinated birds may still develop subclinical infections when exposed to drifted strains or high viral doses. Such “vaccine-breakthrough shedding” is epidemiologically significant, as it allows vaccinated individuals to continue contributing to transmission, preventing meaningful reduction of the flock’s effective reproductive number (R_0_) and enabling within-flock circulation to persist (73, 74).

Mucosal vaccines confer notable advantages in improving herd-level immunity. By inducing sIgA at respiratory and intestinal surfaces, establishing robust TRM networks, and enhancing local innate antiviral readiness, mucosal immunization not only prevents initial infection but also dramatically reduces shedding intensity and duration, thereby lowering transmission probability across the flock. Field monitoring and challenge studies demonstrate that mucosal vaccines reduce environmental viral load, decrease exposure doses for susceptible birds, and consistently drive down flock-level R_0_ (74). Importantly, the rapid mucosal response provides partial preservation of shedding control even against drifted strains—a key limitation of conventional vaccines.

Thus, from the perspective of flock health management and epidemiological containment, mucosal vaccination strategies better align with the core demands of modern poultry production. Their capacity to reduce viral ecological niches, minimize environmental contamination, and interrupt transmission chains positions mucosal vaccines as a promising direction for achieving high-quality herd immunity in future AIV control programs.

## 4 Advances in mucosal vaccine technologies

In recent years, rapid progress in delivery materials, bioengineering, and immunomodulatory technologies has significantly advanced mucosal vaccine development for AIV control. Next-generation mucosal vaccine platforms are increasingly capable of overcoming the physical and immunological barriers of the respiratory mucosa, inducing robust local IgA responses, TRM, and innate antiviral states. These features together establish a strong frontline defense against viral entry and early replication. This section summarizes key technological routes, highlights representative examples from poultry and other respiratory virus vaccines, and discusses critical scientific gaps and future application prospects.

### 4.1 Mucosa-targeted optimization of viral and bacterial vector platforms

Viral and bacterial vector vaccines engineered for mucosal delivery remain among the most mature and widely explored technologies (75). Vectors with natural tropism for the respiratory tract—such as adenovirus, poxvirus, and attenuated Newcastle disease virus (NDV)—can efficiently reach nasal-associated lymphoid tissue (NALT), bronchus-associated lymphoid tissue (BALT), and the Harderian gland following intranasal instillation, spray vaccination, or ocular administration. These vectors rapidly initiate local antigen presentation, IgA secretion, and TRM formation.

For example, adenovirus-based intranasal SARS-CoV-2 vaccines have been shown to induce strong mucosal IgA responses and lung-resident TRM, and similar strategies have demonstrated efficacy in mucosal vaccines using NDV-HA or poxvirus-HA for AIV (76, 77). Optimizing antigen folding, increasing the display density of HA, NA, or M2e, and incorporating mucosal adhesion motifs into vector genomes can enhance antigen retention and immune activation efficiency in the upper respiratory tract. NDV vectors, in particular, are regarded as having high industrial potential for poultry mucosal vaccination due to their low production cost, suitability for mass application, and inherent compatibility with avian respiratory mucosa.

### 4.2 Nanoparticle and advanced biomaterial platforms for enhanced mucosal delivery

The mucus layer, mucociliary clearance mechanisms, and protease-rich environment of the respiratory tract pose significant challenges to antigen stability and retention (78). As a result, developing delivery systems that protect antigens and facilitate their transport across mucosal barriers has become a major focus of mucosal vaccine research.

**Figure 4:**
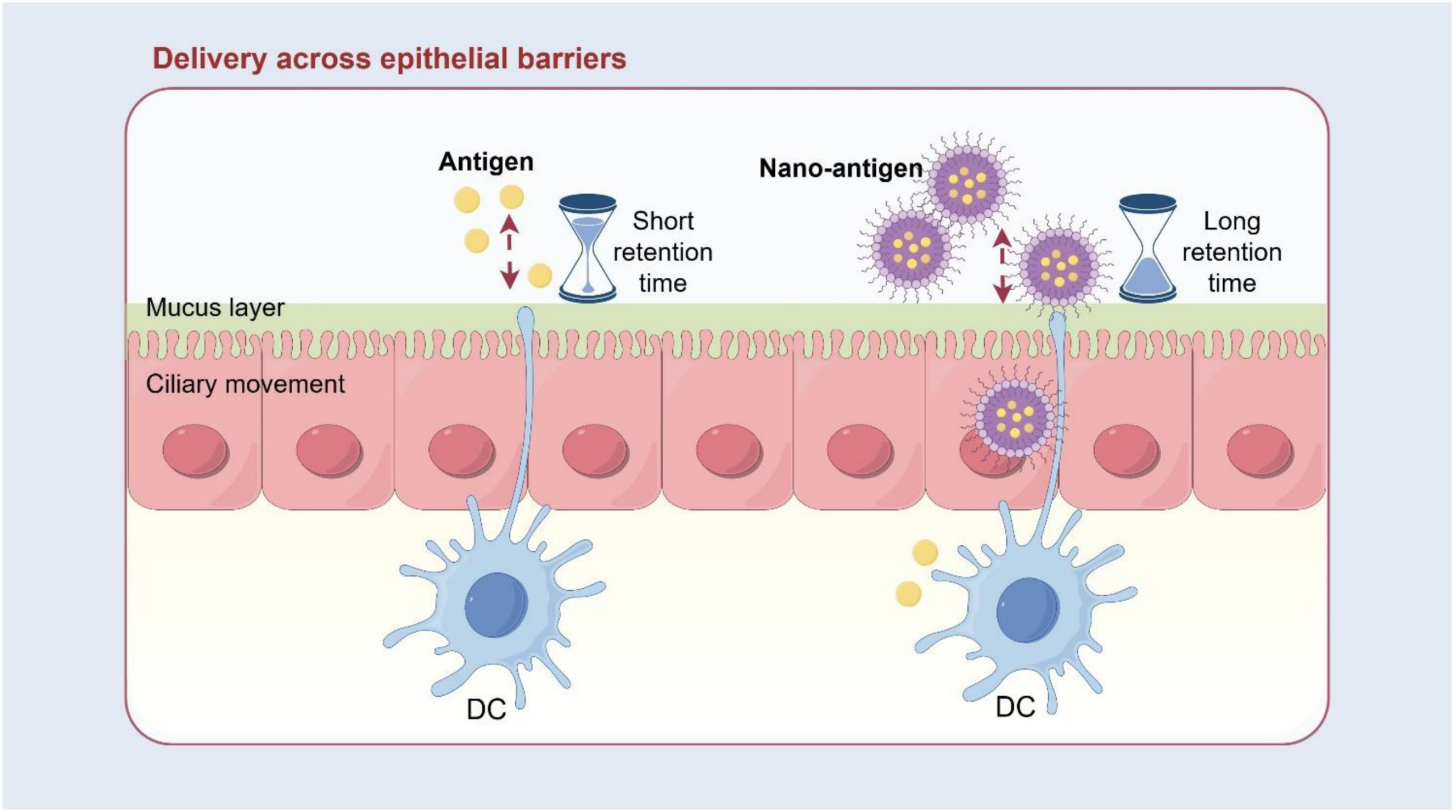
Enhancing antigen stability and mucosal penetration via nanocarriers.

Polymeric nanoparticles (e.g., PLGA, PCL), liposomes, and solid lipid nanoparticles can effectively protect antigens, prolong nasal retention, and improve uptake by M cells and dendritic cells (79, 80) (**Figure 4**). Various PLGA-based HA or M2e nanoparticle vaccines have induced high levels of mucosal IgA and substantially reduced viral shedding in chickens, highlighting their mucosal immunoenhancing potential (81).

Virus-like particles (VLPs), which structurally mimic native virions without replicative capacity, elicit strong innate and adaptive immune responses (82). VLPs constructed from HA trimers, M2e, or multi-subtype antigens have demonstrated broad cross-protection in H5, H7, and H9 models (83). Their successful application in mucosal vaccines for RSV and SARS-CoV-2 further underscores their versatility as next-generation mucosal vaccine platforms.

### 4.3 Emerging advances in RNA vaccines for mucosal immunization

The rise of RNA vaccine technology has opened new avenues for mucosal vaccination in poultry. With improvements in lipid nanoparticle (LNP) chemistry and nucleoside modification, intranasally delivered mRNA–LNP formulations can achieve rapid yet transient antigen expression in respiratory epithelial cells while simultaneously stimulating local interferon responses and activating B/T cell immunity within MALT.

For example, H9 mRNA delivered using the ionizable lipid C12–200 significantly suppressed upper respiratory tract viral replication in chickens (84, 85). The development of lyophilized mRNA–LNP formulations has further improved stability and transportability, making them more suitable for mass spray vaccination in large flocks.

Additionally, H5-HA mRNA constructs incorporating optimized UTR sequences have elicited stronger mucosal IgA responses and prolonged antigen expression in animal models (86–88), indicating that mRNA mucosal vaccines are rapidly moving from experimental validation toward practical application.

### 4.4 Engineered probiotics and plant-based expression platforms for oral mucosal immunization

Engineered probiotics and plant expression systems provide cost-effective and scalable solutions for oral mucosal vaccination in poultry. Probiotics such as *Lactobacillus plantarum* and attenuated *Salmonella* can transiently colonize the gut and present antigens, making them valuable chassis for oral vaccine development (89).

Based on the gut-lung axis theory (90) and evidence that orally administered engineered probiotics can induce respiratory sIgA and reduce viral load in mammalian models (91), the development of an oral engineered probiotic vaccine expressing H9N2 HA holds promise for simultaneously enhancing both intestinal and respiratory sIgA and reducing viral shedding in chickens. This strategy offers a novel approach for the prevention and control of AIV.

### 4.5 Key advances in mucosal adjuvant systems

Innovative mucosal adjuvant systems are essential to overcoming mucosal tolerance and enhancing immune responses. Researchers have developed a range of adjuvants that activate pattern recognition receptors (PRRs) or intracellular innate immune pathways.

TLR3 agonist Poly I:C and TLR9 agonist CpG have significantly enhanced mucosal IgA responses and Th1-biased immunity in multiple influenza mucosal vaccine models (92). STING agonists, including cyclic dinucleotides, strongly promote cytotoxic T cell responses and have been shown to improve cross-protection against highly variable AIV strains (93).

Bacterial outer membrane vesicles (OMVs), enriched with natural PAMPs, serve both as delivery vehicles and immune stimulators. OMV-based vaccines carrying HA or M2e have induced strong mucosal IgA responses in chickens (94). As understanding of NALT, the Harderian gland, and respiratory dendritic cell subsets deepens, targeted adjuvant strategies—such as cationic or mucoadhesive nanoparticles—are emerging as promising tools to enhance mucosal vaccine precision.

Mucosal vaccine technology is advancing toward greater efficiency, safety, and precision. From viral vectors, nanoparticles, and RNA platforms to engineered probiotics and novel adjuvant systems, diverse strategies are being integrated to provide comprehensive immunological protection against AIV. With continued improvements in understanding avian respiratory mucosal architecture and antigen delivery dynamics, mucosal vaccines are poised to become a central technology for combating highly variable AIV strains and enabling large-scale, low-cost, and sustainable flock immunization.

## 5 Global progress in mucosal influenza vaccines

Mucosal vaccines have emerged as a rapidly advancing immunization strategy in recent years, owing to their ability to elicit robust local immune responses at the first barrier of the respiratory tract. Importantly, mucosal vaccines are no longer confined to conceptual or early exploratory stages; several products have already entered clinical use worldwide and have demonstrated favorable safety profiles and strong acceptance, particularly among children and adolescents (**Table 1**). As the risk of AIV continues to rise, mucosa-targeted vaccines against highly pathogenic AIVs are gaining increasing attention and show promising application prospects. In the future, such vaccines are expected to be more widely implemented in both public health and agricultural settings, providing a forward-looking immunological shield against emerging and re-emerging avian-origin influenza threats.

**Table 1:** Overview of clinically available mucosal influenza vaccines.

Vaccine	Type/Route of administration	Target population	Regulatory/development status
FluMist/FluMist Quadrivalent (AstraZeneca/MedImmune)	Intranasal live attenuated influenza vaccine (LAIV)	Humans (seasonal influenza)	FDA-approved in the United States; indicated for individuals aged 2–49 years; under consideration for use in children and adolescents (2–19 years) in Japan
Chinese Intranasal LAIV “Ganwu^®^” (Changchun BCHT Biotechnology)	Intranasal live attenuated influenza vaccine (LAIV)	Humans (seasonal influenza, China)	Licensed in China; indicated for children aged 3–17 years; broadly used in school- and community-based immunization
LUNAR-H5N1 Self-Amplifying mRNA Vaccine (Arcturus Therapeutics)	Self-amplifying mRNA (sa-mRNA) platform; potentially compatible with mucosal delivery	Humans (highly pathogenic avian influenza H5N1)	Entered Phase I clinical trial; designed as a rapid-response vaccine for emerging zoonotic H5N1 threats
Intranasal H5N1 Viral-Vector Vaccine (HKU/CVVT)	Live attenuated viral-vector vaccine; intranasal administration	Animal models; future human pandemic preparedness	Demonstrated strong mucosal IgA and localized T-cell responses in preclinical studies; targets highly pathogenic H5N1
Broad-Spectrum Avian Influenza Candidate Vaccines (DIOSynVax)	Synthetic antigen platform (protein/DNA), with designs compatible with mucosal delivery	Humans (potential); poultry	Preclinical stage; aims to provide cross-subtype, universal-like protection

### 5.1 Licensed intranasal live attenuated influenza vaccines

Intranasal live attenuated influenza vaccines remain the most established mucosal immunization platform. FluMist/FluMist Quadrivalent (AstraZeneca/MedImmune), the most widely used intranasal seasonal influenza vaccine globally, mimics natural infection in the nasal cavity and elicits both robust mucosal and systemic immunity (https://www.fda.gov/media/160243/download) (95). It has been approved by the U.S. FDA for use in children and adolescents, providing strong real-world evidence for the feasibility and effectiveness of mucosal vaccination.

In China, the “Ganwu^®^” intranasal LAIV (Changchun BCHT Biotechnology) represents the first approved mucosal influenza vaccine in the country. It is indicated for individuals aged 3–17 years and has demonstrated excellent compliance and safety in school-based and regional immunization programs, further promoting the translation of mucosal vaccines into public health practice (96, 97).

### 5.2 Mucosal vaccine development targeting highly pathogenic avian influenza

With the increasing threat posed by zoonotic influenza viruses such as H5N1 (98), mucosal vaccines have become a strategic focus owing to their capacity to block viral replication and transmission at the upper respiratory tract.

The LUNAR-H5N1 self-amplifying mRNA vaccine developed by Arcturus Therapeutics has entered Phase I clinical trials (ClinicalTrials.gov NCT05745519). The sa-mRNA platform enables rapid antigen design and high protein expression at low doses (99), and future integration with mucosal delivery technologies may further enhance localized protection. The intranasal H5N1 viral vector vaccine developed by the University of Hong Kong and CVVT has shown strong induction of secretory IgA and mucosal T-cell responses in animal models, conferring significant protection against upper respiratory tract infection (100). This candidate vaccine holds promise for emergency preparedness and provides a foundation for the future deployment of mucosal vaccines against HPAIVs.

### 5.3 Broad-spectrum and novel antigen-designed mucosal vaccine candidates

Research groups such as DIOSynVax are developing broad-spectrum influenza vaccines based on synthetic antigen platforms (protein/DNA), with certain antigen configurations compatible with mucosal delivery systems (101). These vaccines aim to induce cross-protective immunity across multiple subtypes and feature advantages in structural tunability and rapid updating. Although still in preclinical stages, they represent an important technological reserve for potential multi-subtype circulation or emergent influenza outbreaks.

### 5.4 Outlook

Overall, mucosal influenza vaccines are transitioning from traditional LAIVs toward diversified platforms including mRNA, viral vectors, and synthetic antigens, and are becoming an important emerging direction in influenza vaccine development. Licensed intranasal vaccines have already demonstrated clear advantages in safety, compliance, and population-level protection. Next-generation mucosal candidates hold promise for enhanced control of rapidly evolving and cross-species influenza viruses. With ongoing advances in mucosal delivery materials, antigen engineering, and thermostability, mucosal vaccines are expected to play an increasingly significant role in seasonal influenza prevention and pandemic preparedness. Notably, mucosal vaccines targeting avian influenza may achieve broader application in the near future as technology maturation accelerates.

## 6 Prospects for the application of mucosal influenza vaccines in the poultry industry

### 6.1 Industrial demand for mucosal vaccination in modern poultry production

Mucosal vaccines have emerged as a rapidly advancing immunization strategy in recent years, offering features that better align with the operational needs of large-scale avian influenza control compared with traditional injectable vaccines. With the expansion of intensive farming and rising labor costs, the poultry industry increasingly requires vaccines capable of achieving rapid mass immunization, reducing handling-related stress, and effectively lowering viral shedding.

Importantly, mucosal vaccination is not merely a conceptual or experimental approach but has already been implemented in poultry production systems in several countries. Spray- and drinking-water–administered vaccines, particularly those based on Newcastle disease virus (NDV) or fowlpox virus (FPV) vectors, have been widely used in commercial settings for respiratory viral diseases, demonstrating both feasibility and operational advantages under field conditions. These real-world applications highlight the practicality of mucosal immunization strategies in high-density farming environments.

Under these conditions, mucosal vaccines—owing to their unique immune-activation mechanisms, proven field applicability, and ease of administration—are becoming a central component of future integrated avian influenza prevention and control programs, with strong potential for broader adoption across diverse poultry production systems.

### 6.2 Economic and management advantages of mucosal vaccines in large-scale farms

From an economic and management perspective, large commercial farms are highly cost-sensitive. Traditional intramuscular vaccination typically requires substantial numbers of skilled workers, and labor plus stress-associated losses can account for 40–60% of total immunization costs (WOAH Poultry Vaccination Guidelines), which is particularly detrimental to the broiler industry characterized by short production cycles and narrow profit margins.

Although the intrinsic cost of mucosal vaccines may be relatively high during early development and initial deployment—due to formulation optimization, specialized delivery systems, and regulatory investment—these costs tend to decline substantially as manufacturing processes mature and large-scale production is established. In contrast, mucosal vaccination via spray, drinking-water administration, or eye–nose drops significantly reduces labor requirements and improves operational efficiency, enabling the immunization of tens of thousands of birds within minutes.

Moreover, by avoiding the stress associated with handling and catching birds, mucosal vaccination reduces mortality, improves daily weight gain, and enhances overall production performance, thereby offsetting initial vaccine costs and offering clear economic advantages in long-term, large-scale poultry operations.

### 6.3 Mucosal vaccine platforms suitable for industrial application

From the perspective of technological maturity and vaccine platform development, mucosal vaccine types currently suitable for industrial application include recombinant viral-vector vaccines based on NDV and FPV, sprayable attenuated AIV live vaccines, and rapidly advancing nanoparticle-delivered subunit vaccines. NDV and FPV vector platforms feature mature manufacturing processes, stable antigen expression, and compatibility with multivalent antigen design; they have already been integrated into routine vaccination programs in several countries and are particularly advantageous for high-density broiler farms. Sprayable attenuated AIV vaccines provide strong systemic and mucosal immunity and are effective in rapidly reducing viral shedding. Meanwhile, novel mucosal subunit vaccines delivered via chitosan, PLGA, or lipid nanoparticles offer excellent safety profiles and efficient antigen presentation, representing highly promising candidates for high-value poultry populations such as layers and breeders.

### 6.4 Immunological advantages of mucosal vaccination for transmission blocking

With the continued expansion of large-scale and intensive poultry production, establishing a mucosal-immunity-centered “frontline blocking” immunization strategy will be critical for the long-term stable control of avian influenza. Unlike injectable vaccines that primarily induce humoral responses, mucosal vaccines can rapidly elicit local IgA, TRM, and epithelial-associated innate immune networks at the site of viral entry. This enables strong early containment of viral replication and interruption of within-flock transmission—an essential advantage for reducing the hidden economic losses associated with large-scale production. For low-pathogenic AIV, which often causes subclinical reductions in egg production or body-weight gain, mucosal immunization can substantially improve productivity by reducing subclinical infections, further highlighting its industrial value.

### 6.5 Future directions and cost-effective mucosal vaccine development

Looking ahead, mucosal vaccine platforms characterized by low production costs, stable immunogenicity, and scalable manufacturing hold the greatest promise. NDV-vector vaccines—due to their moderate replication levels and ease of spray administration—are expected to maintain dominance in the broiler industry. Nanoparticle-based vaccines, with their broad-spectrum potential and strong safety profiles, are well suited for long-term immunization programs in layers and breeders and are likely to achieve large-scale adoption within the next 5–10 years. Additionally, oral vaccines delivered via engineered lactic acid bacteria represent an exceptionally low-cost and logistically convenient approach that may achieve early breakthroughs in developing countries, though further optimization of antigen stability and dose control is still required.

Overall, the industrial application of mucosal vaccines represents a shift in poultry immunization strategies from “therapeutic-type immunity” to “blocking-type immunity.” This transformation aligns closely with modern farming goals of antimicrobial reduction, cost minimization, improved animal welfare, and enhanced biosecurity. With continued progress in vector design, antigen engineering, and mucosal delivery technologies, mucosal vaccines are expected to occupy a central position in global avian influenza immunization programs and may progressively replace traditional injectable vaccines in certain settings, providing more efficient, economical, and forward-looking solutions for disease control in large-scale poultry production.

## 7 Future research directions and challenges

### 7.1 Species-specific differences in avian mucosal immunity

**Figure 5:**
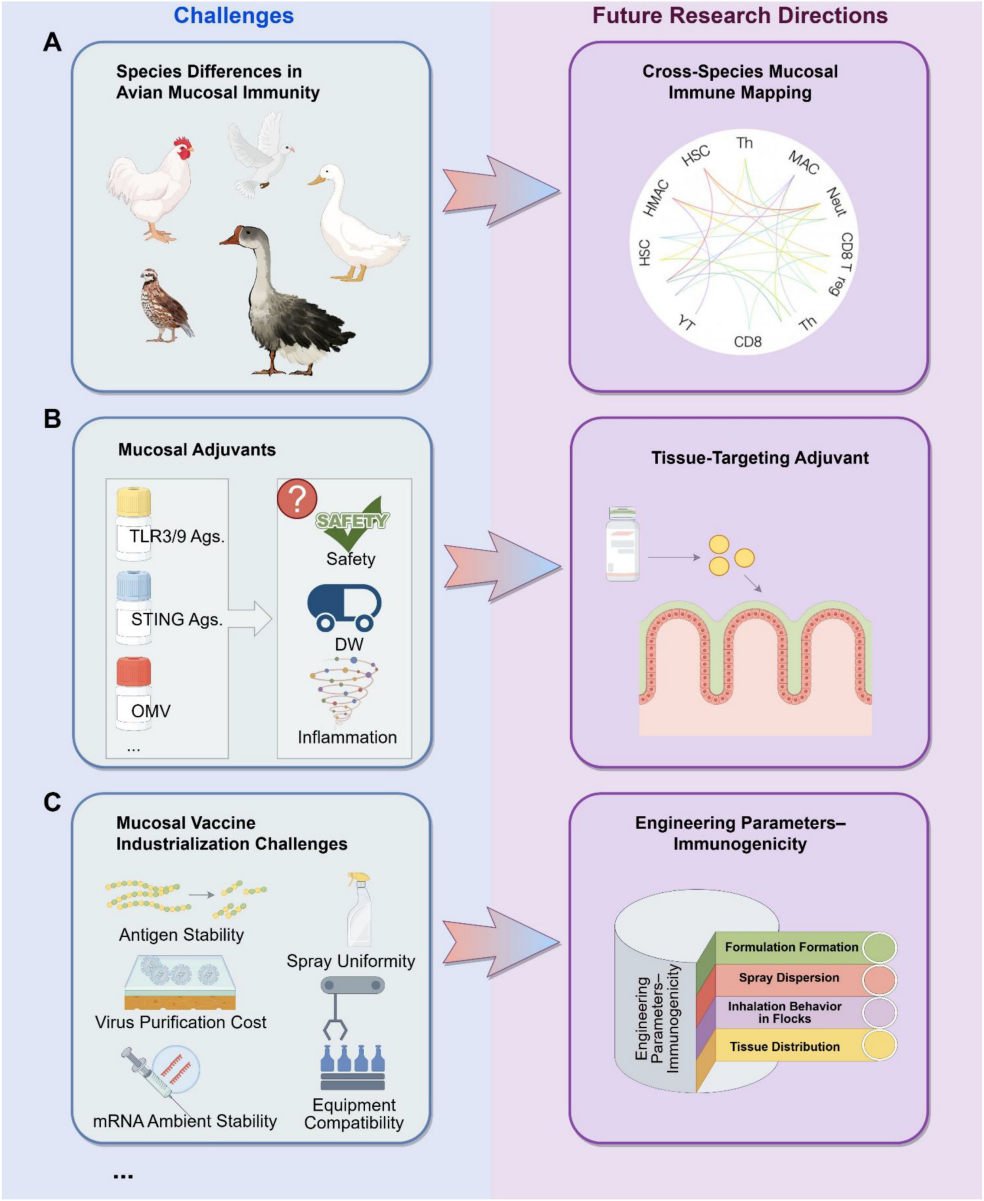
**(A)** Species-specific differences in avian mucosal immunity. **(B)** Bottlenecks in mucosal adjuvant development. **(C)** Engineering and formulation challenges in industrial-scale manufacturing.

Although mucosal vaccines exhibit clear advantages in avian influenza control, their translation into mature products and large-scale application is constrained by significant knowledge gaps in avian mucosal immunology. Marked interspecies differences exist in nasal epithelial architecture, mucus composition, M-cell distribution, and the development of BALT and Harderian glands (102). For example, chickens possess a highly developed Harderian gland that plays a central role in IgA secretion and local T-cell responses, whereas ducks and geese have mucosal surfaces adapted to humid environments with potentially distinct antigen retention and uptake mechanisms (103). Most mucosal vaccine platforms are still developed primarily in chicken models, resulting in inconsistent protection or failure when applied across species. Future work should focus on constructing cross-species mucosal immune atlases, developing standardized evaluation systems, and designing delivery materials and adjuvant platforms adaptable to multiple avian species (**Figure 5A**).

### 7.2 Bottlenecks in mucosal adjuvant development

The development of effective mucosal adjuvants remains a major bottleneck limiting the industrialization of mucosal vaccines. Current candidates—including TLR3/9 agonists, STING agonists, outer membrane vesicles (OMVs), cholesterol derivatives, and biodegradable polymer particles—lack systematic assessment regarding safety, dose windows, inflammatory profiles, and long-term impacts. Unlike injectable adjuvants, mucosal adjuvants must simultaneously enhance immunity, protect the epithelial barrier, maintain productivity, and remain stable under spray or intranasal administration. Future innovation may rely on tissue-targeting adjuvants tailored to specific mucosal sites such as the nasal cavity, Harderian gland, trachea, or intestinal mucosa (**Figure 5B**).

### 7.3 Unresolved mechanisms underlying long-term IgA and TRM maintenance

The long-term maintenance of mucosal IgA and TRM is fundamental to the advantages of mucosal vaccination, yet remains poorly characterized in avian species. Avian mucosal epithelia undergo rapid turnover, high regenerative activity, and significant variation with age and environmental conditions, all of which influence TRM residency and IgA stability. Mechanistic insights from mammals cannot be directly extrapolated to birds, where empirical evidence is largely lacking. Future studies should address TRM metabolic requirements, the regulatory roles of local cytokines (e.g., IL-17, IL-10, TGF-β), microenvironmental cues controlling IgA^+^ B-cell differentiation and localization, and the impact of farming conditions (temperature, humidity, ammonia, dust) on mucosal immune homeostasis.

### 7.4 Engineering and formulation challenges in industrial-scale manufacturing

Industrial translation of mucosal vaccines faces significant formulation and engineering challenges. These include antigen shear stability during spraying, aerosolization or administration via drinking water; consistency and cost-effectiveness of nanoparticle or viral-vector purification; thermostability of mRNA or VLP formulations; and control of spray uniformity and actual dose uptake by flocks. Practical constraints within farms—such as mixing logistics and equipment compatibility—further complicate deployment. Future efforts should aim to build an integrated evaluation pipeline linking formulation properties, spray dynamics, avian inhalation behavior, and tissue distribution, enabling a systems-level correlation between engineering parameters and immunological outcomes (**Figure 5C**).

### 7.5 Influence of flock structure and environmental factors on mucosal vaccine performance

In real-world poultry production, vaccine performance depends not only on individual immune responses but also on flock-level structure, environmental conditions, and co-infections (104). High-density housing may alter mucosal immune thresholds; vertical stratification of birds contributes to uneven spray distribution; ammonia and particulate matter can damage epithelial barriers; and co-infections (e.g., *E. coli*, IBV) may reshape the mucosal immune microenvironment (105). These complexities hinder the direct translation of laboratory-generated efficacy data to field conditions. Establishing “immune–environment–microbiota interaction models” applicable across farming scenarios will be essential for optimizing mucosal vaccine deployment.

### 7.6 System-level mucosal immunology enabled by emerging technologies

Driven by the rapid development of gene editing, single-cell and spatial transcriptomics, microbial and metabolic profiling, AI-based vaccine design, and smart farming technologies, mucosal vaccine research is transitioning from isolated product development to a system-level mucosal immunology framework. Key future priorities include precision mucosal antigen design, microenvironment-targeted delivery strategies, computational immune modeling and AI-driven optimization, and integrated multi-platform vaccination strategies. Through such multidisciplinary approaches, mucosal vaccines are expected to become central components of avian immunoprophylaxis, enabling more precise and durable control of both highly pathogenic and low pathogenic AIVs, and ultimately reducing subclinical infections and economic losses.

## 8 Conclusion

Mucosal vaccination offers a fundamentally different and complementary strategy for avian influenza control by strengthening immune protection at the sites of viral entry. By effectively reducing viral shedding and interrupting transmission, mucosal vaccines address key limitations of conventional injectable vaccines at the population level.

Ongoing advances in mucosal antigen design, delivery systems, and adjuvant technologies are improving feasibility for large-scale application in poultry production. Importantly, beyond enhancing flock health, mucosal immunization contributes to lowering the risk of cross-species transmission, underscoring its relevance within integrated avian influenza control and One Health frameworks.

With continued progress in avian mucosal immunology and vaccine engineering, mucosal vaccines are poised to become an essential component of sustainable and forward-looking avian influenza prevention strategies.
